# Supplementary material for: A20 restriction of nitric oxide production restores macrophage bioenergetic balance
Source: bioRxiv. 2025 Oct 29:2025.10.26.684676. Preprint. [Version 2] doi: 10.1101/2025.10.26.684676 (PMC12636326; doi:10.1101/2025.10.26.684676)

Supplementary Figure 1: Restoration of M2 repolarization by iNOS inhibitör.
Flow cytometry analysis of CD206 (M2 marker) expression on A20^{tiKO} and WT BMDMs after 24 hours of LPS or LPS+1400w, followed by 24 hours of IL-4.
Note reduced induction of CD206 expression by IL-4 in A20^{tiKO} BMDMs compared with WT cells, and normalization of CD206 expression in A20^{tiKO} BMDMs with iNOS inhibitor 1400w.
Data are representative of two independent experiments.

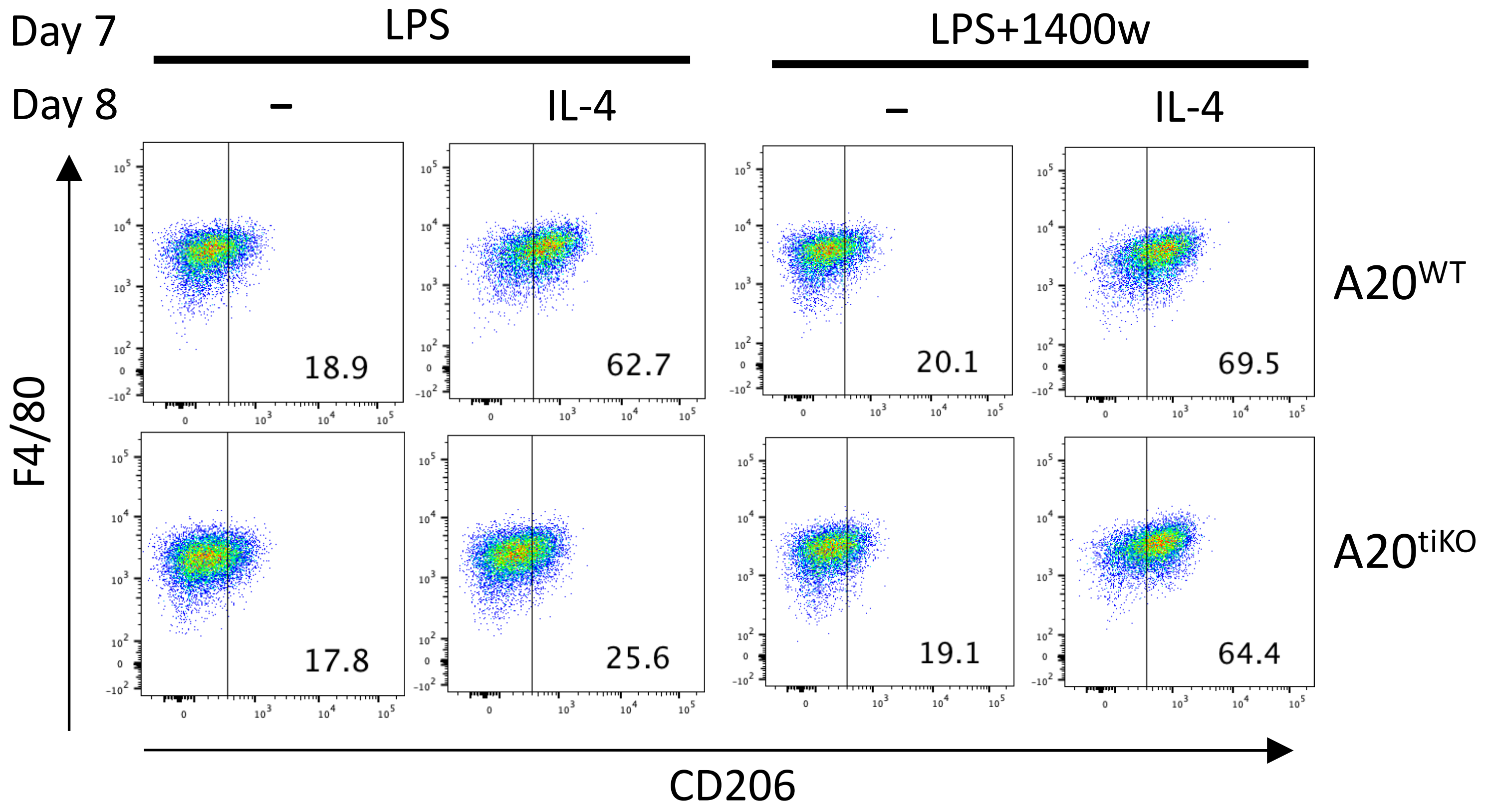

Supplement: Supplement 1 [file media-1.pdf]
