# Supplementary material for: A20 restriction of nitric oxide production restores macrophage bioenergetic balance
Source: bioRxiv. 2025 Oct 29:2025.10.26.684676. Preprint. [Version 2] doi: 10.1101/2025.10.26.684676 (PMC12636326; doi:10.1101/2025.10.26.684676)

Supplementary Figure 2: Immunoblot of iNOS and A20 protein expression in BMDMs from indicated genotypes. Tubulin expression show below as loading control. Data are representative of three independent experiments.

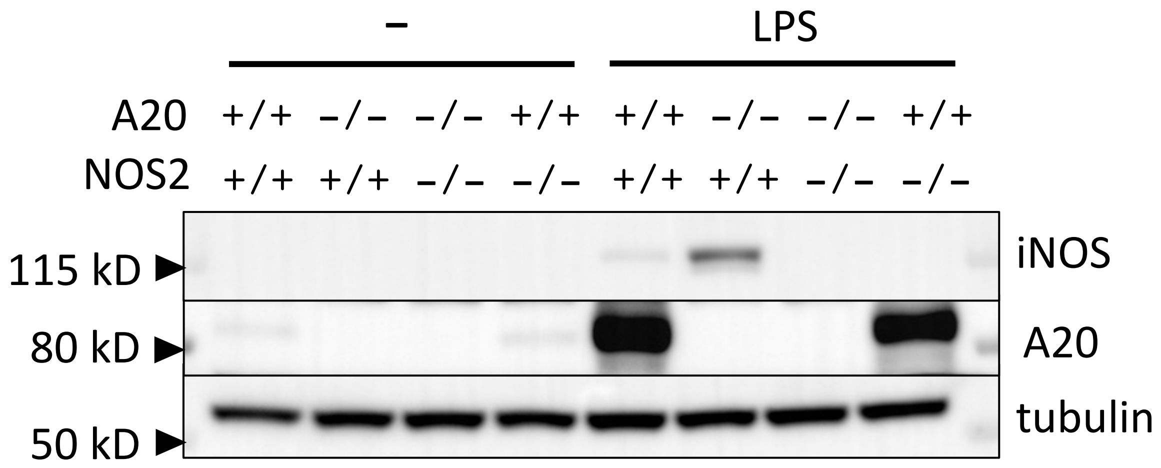

Supplement: Supplement 2 [file media-2.pdf]
