# Supplementary material for: A20 restriction of nitric oxide production restores macrophage bioenergetic balance
Source: bioRxiv. 2025 Oct 29:2025.10.26.684676. Preprint. [Version 2] doi: 10.1101/2025.10.26.684676 (PMC12636326; doi:10.1101/2025.10.26.684676)

Supplementary Figure 3: Flow cytometric measurement of ROS/RNS in indicated genotypes after 1400w iNOS inhibitor treatment at indicated times.
Note that iNOS inhibition normalizes ROS/RNS in LLPS stimulated A20tiKO cells.
Mean values ± SD are shown. **p<0.01 by unpaired two-tailed t test was used.
Data are representative of three independent experiments.

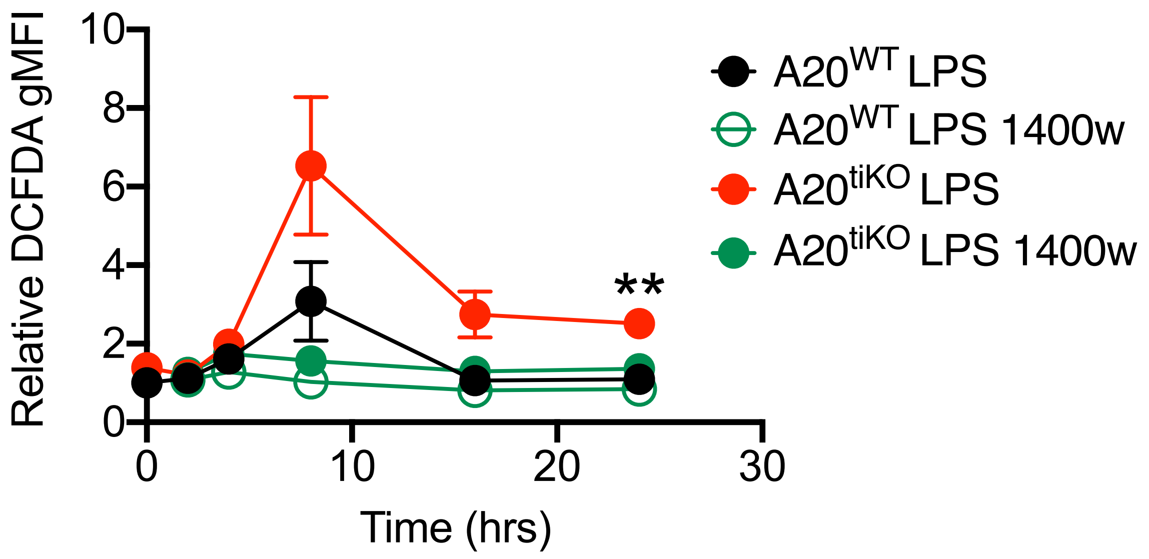

Supplement: Supplement 3 [file media-3.pdf]
